# Healthy Vaginal Microbiota and Influence of Probiotics Across the Female Life Span

**DOI:** 10.3389/fmicb.2022.819958

**Published:** 2022-04-08

**Authors:** Liisa Lehtoranta, Reeta Ala-Jaakkola, Arja Laitila, Johanna Maukonen

**Affiliations:** IFF Health & Biosciences, Kantvik, Finland

**Keywords:** vaginal, microbiota, mycobiota, lactobacilli, probiotics, health, dysbiosis

## Abstract

Vaginal microbiota plays a central role in women’s health and reproduction. Vaginal microbiota is dynamic and shaped by hormonal shifts in each stage of a woman’s life from pre-puberty to postmenopause. Current research has mainly focused on vaginal bacterial and fungal members of the community and emphasized their role in disease. However, the impact of balanced vaginal microbiota on health and its interaction with the host is yet poorly understood. High abundance of vaginal lactobacilli is most strongly associated with health, but the concept of health may vary as vaginal dysbiosis may be asymptomatic. Furthermore, there is a lot of variation between ethnic groups in terms of dominating vaginal bacteria. Probiotic lactobacilli could be a safe and natural means to balance and maintain healthy vaginal microbiota. Research evidence is accumulating on their role in supporting women’s health throughout life. This review describes the current literature on vaginal microbiota, the major factors affecting its composition, and how the communities change in different life stages. Furthermore, we focused on reviewing available literature on probiotics and their impact on vaginal microbiota and health.

## Introduction

In the past decade, exploration of human microbiota has increasingly focused on vaginal microbiota (VMB) composition, diversity, and its impact on health, reproduction, and disease. VMB is known to consist of various bacteria, viruses, archaea, fungi, and protozoa (Ursell et al., 2012; Dekaboruah et al., 2020). Nevertheless, the research has mainly focused on bacterial and fungal members in the vaginal community and their association with women’s health. Typically, VMB is described in the context of vaginal infections or dysbiosis, which occurs, e.g., *via* antibiotic use or lifestyle changes. Most common infections and conditions include vulvovaginal candidiasis (VVC) and bacterial vaginosis (BV), respectively [reviewed by van de Wijgert and Jespers (2017), De Bernardis et al. (2018)]. BV is in turn associated with urinary tract infections (UTIs), increased risk of infertility, fallopian tube (uterine tube) inflammation, adverse pregnancy outcomes, and preterm birth (PTB) [Hootan et al., 1989; Hay et al., 1994; Hillier et al., 1995; Koumans et al., 1999; Hillerbrand et al., 2002; reviewed by van Oostrum et al. (2013)]. Moreover, dysbiosis in the vaginal tract is associated with sexually transmitted infections such as human immune deficiency virus (HIV), human papillomaviruses (HPV), herpes, chlamydia, and gonorrhea [reviewed by Gupta et al. (2019)].

In contrast to the gut (Eisenstein, 2020), defining the characteristics of healthy microbiota in the vaginal tract seems to be less challenging, as one of the hallmarks of a healthy vaginal tract is low bacterial diversity and high abundance of vaginal lactobacilli. These lactobacilli play a fundamental role in interacting with the host innate immune system, and research shows that low abundance of lactobacilli increases the risk of several vaginal infections (Amabebe and Anumba, 2018b). Lactobacilli for instance produce lactic acid which creates an acidic microenvironment and thus prevents the overgrowth of potentially harmful bacteria. However, the definition of healthy VMB is not straightforward. For example, vaginal dysbiosis is not always presented with symptoms and not all species of vaginal lactobacilli are equally protective (Mitchell et al., 2017; Smith and Ravel, 2017). VMB varies among ethnic groups, where healthy/balanced state is maintained with more diverse VMB composition (e.g., among Black or African American) (Zhou et al., 2007). Furthermore, hormonal fluctuations shape the microbial community even on a daily or monthly basis (Eschenbach et al., 2000). In addition, the ways on how the healthy VMB is shaped in women at different stages of life have not been comprehensively characterized.

Vaginal dysbiosis and associated infections affect all women during their lifetime by causing discomfort and are the most common reasons why women seek medical care [reviewed by van de Wijgert and Jespers (2017), Peebles et al. (2019)]. Unfortunately, antibiotics and antifungal medicines to treat BV and candidiasis are not always optimal in efficacy as bacteria and fungi are able to resist these treatments by forming biofilms or they have naturally acquired resistance (Swidsinski et al., 2008, 2015; Bhattacharya et al., 2016, 2020). Antibiotics may also disrupt endogenous lactobacilli promoting BV and potentially candidiasis recurrence (Bradshaw and Sobel, 2016; Verwijs et al., 2020). Therefore, rigorous research is underway to identify more effective and natural solutions for balancing VMB and maintaining healthy vaginal environment. Well-characterized probiotic bacteria, which are typically lactobacilli, could be applied for maintaining healthy VMB or restoring normal microbiota after antibiotic treatment. Indeed, several systematic reviews and meta-analyses suggest that probiotic use could be potential, e.g., in helping to relieve BV (Li et al., 2019; Wang et al., 2019) or VVC (Xie et al., 2017; Jeng et al., 2020).

The main objectives of this review were to provide first an overview of VMB and major factors affecting the composition. Secondly, we focused on identifying and describing the key changes in VMB throughout different life stages of women and discussed the impact of these changes in health. As probiotics are targeted for maintaining and promoting health and healthy microbiota, we also explored the probiotic’s role on VMB and women’s health across the lifespan.

## Overview of Vaginal Microbiota and Factors Affecting the Composition

Advancements in the next-generation sequencing technologies and bioinformatic tools have rapidly increased our understanding of all members in the VMB community—bacteria, viruses, archaea, fungi, and protozoa, of which identification was previously difficult with other methods, such as culturing (Hong et al., 2016; Ceccarani et al., 2019). However, if there are no cultured isolates for a bacterium identified with molecular techniques, full understanding of the potential impact/mechanism of action of how the specific bacterium may influence vaginal health and/or disease may not be fully comprehended. In these cases, culturomics approach, using dozens of different culture media and conditions, could prove useful (Lagier et al., 2012). Moreover, advances in culturomics have enabled isolation and identification of hundreds of new human-associated bacteria. In practice, the number of cultured human-associated bacterial species increased from 2,776 in 2018, to 3,253 in 2020 (Diakite et al., 2021). Within these new genera/species were also many that originated from vaginal isolates, such as *Vaginimicrobium propionicum*, a novel propionic acid bacterium isolated from a healthy human’s vaginal discharge (Diop et al., 2020) and *Janibacter massiliensis* isolated from vaginal discharge of a bacterial vaginosis patient (Maaloum et al., 2019).

Thus, what was previously regarded as relatively simple microbial community has now been revealed to be in fact dynamic and complex. Each woman has her own unique VMB composition, which fluctuates over time and is affected, e.g., by diet, lifestyle, hormones, genetics, and age [reviewed by Farage and Maibach (2006); Gupta et al. (2019)]. Here we focused on the bacterial and fungal members of the community as these are the most prevalent microbes associated with vaginal health.

### Bacteria and Fungi

Vaginal microbiota composition in humans is relatively unique when compared with other mammals, including non-human primates. For instance, lactobacilli typically dominate 70% of the human VMB community, while in other mammals, lactobacilli comprise only low percentages of the total VMB composition (Stumpf et al., 2013; Miller et al., 2016). This in turn is associated with low glycogen and lactic acid levels. Consequently, other mammals have vaginal pH closer to neutral.

Advanced molecular detection methods utilizing 16S rRNA gene sequencing have enabled clustering of the human vaginal bacterial community into specific community state types (CSTs) (Smith and Ravel, 2017; France et al., 2020). CST I, II, III, and V are dominated by *Lactobacillus crispatus*, *Lactobacillus gasseri*, *Lactobacillus iners*, and *Lactobacillus jensenii*, respectively. CST IV includes higher abundance of strictly anaerobic bacteria such as species from genera *Prevotella, Dialister, Atopobium, Gardnerella, Megasphaera, Peptoniphilus, Sneathia, Eggerthella, Aerococcus, Finegoldia*, and *Mobiluncus* and is characterized by higher diversity and evenness (Ravel et al., 2011; Gajer et al., 2012; France et al., 2020). CST IV can be further divided into subgroups: CST IV-A containing a proportion of *L. iners* and strict anaerobes, and CST IV-B containing BV-associated bacterial species (BVAB) (bacteria in the order Clostridiales) (Anahtar et al., 2018; France et al., 2020). CST IV-C is characterized by the abundance of a diverse array of facultative and strictly anaerobic bacteria and can be further divided into five sub-categories (France et al., 2020). Overall, CST I, II, and V are most often associated with health, whereas predominance of CST IV can manifest clinically as BV or aerobic vaginitis (AV). Interestingly, CST III has been associated with both health and dysbiosis, and often with BV.

The role and impact of fungi overall are much less understood compared to bacteria. Although fungi have an important role in maintaining homeostasis in the human body, and fungi contribute to our immune defense, much less is known about the role of fungi in healthy vagina throughout the lifespan. However, knowledge on the fungal community structure and distributions is rapidly increasing (Nash et al., 2017; Chee et al., 2020). High diversity is observed within and across individuals (Nash et al., 2017; Forbes et al., 2019), but this diversity is significantly lower than the bacterial diversity (Nash et al., 2017) as well as greater unevenness when compared to the bacterial communities (Raimondi et al., 2019). However, fungi comprise a smaller portion of human microbial sequences compared to bacteria (Kong and Morris, 2017). The fungal community, including yeasts and filamentous fungi colonizing the lower female reproductive tract, is referred to as vaginal mycobiota (Bradford and Ravel, 2017). Vaginal fungi mainly belong to Ascomycota and Basidiomycota (Drell et al., 2013; Bradford and Ravel, 2017; Chee et al., 2020). The predominant fungal genera in the genitourinary tract (vagina) include *Candida*, *Cladosporium, Pichia, Aspergillus*, and *Rhodotorula* (Drell et al., 2013; Lai et al., 2019). Notably, many studies reported unspecified fungal sequences which were not taxonomically identified during that time (Drell et al., 2013). In total, approximately 390 different fungi have been associated with human skin, vagina, oral cavity, and gut samples so far (Gouba and Drancourt, 2015). Changes in bacterial communities due to the dysbiosis is known to increase opportunities for fungal growth and for opportunistic pathogens.

*Candida* is the predominant genus in the vaginal mycobiota, and several species have been detected including *Candida albicans, C. alimentaria, C. dubliensis, C. glabrata, C. krusei, C. parapsilosis*, and *C. tropicalis* (Drell et al., 2013; Ward et al., 2018; Chee et al., 2020). Interestingly, vaginal colonization with *Candida* spp. seems to occur more commonly in women with a lactobacilli-dominated VMB than in women with dysbiosis (van de Wijgert et al., 2014). The research so far has been mainly focused on *C. albicans*, which is the best-known member of the vaginal mycobiota (Bradford and Ravel, 2017). It is an opportunistic pathogen causing VVC. However, *C. albicans* has been reported to be the dominant fungal species in asymptomatic non-pregnant women and it colonizes 20% of women (Drell et al., 2013; Ameen et al., 2017; Chee et al., 2020). Our recent study supported the previous findings that *C. albicans* and/or other *Candida* spp. were detectable in approximately 17% of samples derived from healthy women (Lehtoranta et al., 2021). However, a lot of variation exists between the studies as Drell et al. (2013) reported that *Candida* spp. could be detected in 65% of asymptomatic healthy women.

### Major Factors Affecting the Composition

**FIGURE 1:**
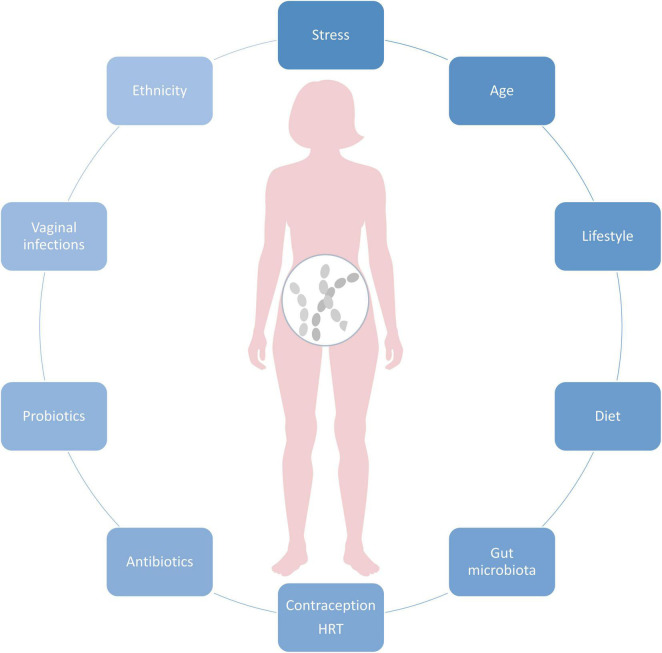
Key factors affecting vaginal microbiota composition.

Vaginal microbiota composition is highly dynamic and affected by age, ethnicity, and physiological factors, such as monthly hormonal fluctuations and the immune system. Vaginal infections, medications, as well as probiotics shape the VMB, as well as lifestyle and diet (Figure 1). In the below chapters we focus on major known modulators of VMB.

#### Ethnicity

Genetic heritage plays a role determining VMB composition as the CSTs are known to vary among ethnic groups. In general, lactobacilli dominate the VMB among the Asian and White women, whereas Black or African-American and Hispanic women are more likely to be dominated with diverse VMB, containing several BV-associated species, such as *Gardnerella vaginalis*, BVAB1, BVAB2, *Atopobium vaginae*, *Megasphaera* spp., *Sneathia* spp., and *Prevotella* spp. (Ravel et al., 2011; Fettweis et al., 2014; Borgdorff et al., 2017; Gupta et al., 2017) and CST IV-A or B (France et al., 2020). Women with black ethnicity are considerably more likely to be diagnosed with BV and PTB (Fettweis et al., 2014). Interestingly, *L. iners*-dominated VMB (CST III) is prevalent in VMB of women in all ethnic groups [reviewed by Petrova et al. (2017)]. Limited evidence exists for vaginal *Candida* species and their prevalence among ethnic groups (Wei et al., 2010).

#### Menstrual Cycle

Reproductive hormones and especially estrogen play a key role in shaping the VMB composition throughout life from the onset of puberty, during reproductive years toward menopause, and postmenopause when the estrogen levels decline [reviewed by Moosa et al. (2020)].

In the vaginal epithelial cells, estrogen increases glycogen storage, which is an important energy source for vaginal lactobacilli affecting their ability to colonize the vaginal tract and produce lactic acid, and which further promotes the ability to grow and thrive in acidic conditions. Therefore, it is evident that estrogen fluctuations throughout menstrual cycle modulates VMB composition (Eschenbach et al., 2000; Srinivasan et al., 2010; Gajer et al., 2012). The highest estrogen levels occur in the follicular phase, which correlates with dominance of lactobacilli and low bacterial diversity (Kaur et al., 2020). Gajer et al. (2012) investigated temporal dynamics of the vaginal bacterial communities in 32 women at reproductive age over 16 weeks (including 4–5 menstrual cycles). The study revealed variation across women in terms of how communities changed over short periods of time. For instance, women with CST IV-B often transitioned to CST III. Likewise, women with CST I (*L. crispatus*) most often transitioned to CST III (*L. iners*), or CST IV-A. Interestingly, CST II (*L. gasseri*) rarely underwent transitioned to other CSTs. The bacterial communities seemed to be less stable during menses and to have low abundance of lactobacilli, although diversity was not necessarily increased. Similarly, in another study with 14 healthy women, Srinivasan et al. (2010) showed that vaginal lactobacilli fluctuated over time. However, at the onset and during menses, the dominance of *L. crispatus* and *L. jensenii* significantly decreased, whereas *G. vaginalis* and *L. iners* increased and subsequently decreased with the end of menstruation. This finding may be potentially explained by the fact that *G. vaginalis* requires iron for growth and could acquire iron from menstrual blood by lysing erythrocytes with vaginolysin (Jarosik et al., 1998; Srinivasan et al., 2010). *L. iners* is also able to grow well in blood agar (Falsen et al., 1999), but not in MRS or Rogosa agar, indicating the potential requirement of iron of this species for growth and thus may explain why the strains favor co-occurrence during menses. Another longitudinal study investigating VMB composition throughout two menstrual cycles reported that *L. crispatus* was the least affected by the menses (Lopes Dos Santos Santiago et al., 2011). With regard to the effects of menstrual cycle on the mycobiota, studies suggest that the estrogen peak during ovulation stimulates the growth of *C. albicans* resulting in higher occurrence of symptoms of *C. albicans* later in the luteal phase (Eschenbach et al., 2000).

Despite the widespread use of hormonal contraceptives during reproductive years (United Nations, Department of Economic and Social Affairs, Population Division, 2019), their effects on VMB communities and the dynamics have not been fully elucidated (Achilles and Hillier, 2013; Anahtar et al., 2018) and there are inconsistent results due to variable contraceptive methods used. However, it seems that there is an association between oral contraceptives and reduced BV (Achilles and Hillier, 2013; Brooks et al., 2017; Anahtar et al., 2018). For yeasts, there is no consensus whether hormonal contraceptives increase or reduce vaginal yeast colonization or their infections (Achilles and Hillier, 2013). A study by Song et al. showed that healthy women not using hormonal contraceptives and women using combined contraceptives had similar periodic fluctuations of VMB that corresponded to stages of the menstrual cycle and high *Lactobacillus* abundance. Then, women with progestin-only contraceptives showed altered periodic fluctuations of VMB and low average abundance of *Lactobacillus* (Song et al., 2020).

#### Lifestyle

Similar to the gut microbiota, different lifestyle factors influence VMB composition [reviewed by Bradford and Ravel (2017); Moosa et al. (2020)]. Here we focus on the most documented factors as well as emerging factors influencing VMB that are a direct result of modernization of the society. Intuitively, personal hygiene practices (i.e., vaginal douching, use of soaps, type of underwear, menstrual protection, and sprays) are the most direct ways to affect VMB composition. Among these, research suggests that vaginal douching seems to be most strongly associated with increased risk of BV/vaginal dysbiosis (Klebanoff et al., 2010; Lewis et al., 2017). In contrast, for the occurrence of *Candida* infection type in VVC, vaginal douching seems to have a low impact (Shaaban et al., 2015). Regarding sexual habits, multiple sexual partners are also a known risk factor for BV/lactobacilli depletion (Beigi et al., 2005; Schwebke and Desmond, 2005).

In addition, smoking is a well-known factor to increase the risk of vaginal dysbiosis and BV (Hellberg et al., 2000; Cherpes et al., 2008; Brotman et al., 2014a) by, e.g., affecting estrogen production (Westhoff et al., 1996) and altering vaginal metabolite production profile, particularly by increasing levels of nicotine and derivatives as well as biogenic amines (Nelson et al., 2018). Similarly, alcohol use is associated with increased BV cases (Francis et al., 2016).

Interestingly, new emerging research indicates that the modernization of the society related to, e.g., increased psychological stress, consumption of processed food rich in fat and carbohydrates, and urbanization has an impact on VMB [Neggers et al., 2007; Thoma et al., 2011b; reviewed by Amabebe and Anumba (2018a); Vargas-Robles et al. (2020)]. Women experience more stress than men (American Psychological Association, 2018) and the effects of stress seem to extend to the vaginal tract. More specifically, research implies that chronic psychosocial stress can influence the balance in the vaginal lactobacilli, potentially through dysregulated immune system and elevated cortisol levels, which further correlate with reduced vaginal glycogen, lower abundance of lactobacilli, elevated vaginal pH, and increased proinflammatory response [reviewed by Amabebe and Anumba (2018a)].

Diet does not only shape the gut microbiota, but the effects are known to extend to the vaginal tract. More specifically, research suggests that healthy diet rich in nutrients and with low glycemic index and lower fat intake could reduce the risk of BV (Neggers et al., 2007; Thoma et al., 2011b). Furthermore, micronutrient intake, particularly increased folate, vitamin A, and calcium could decrease BV risk (Neggers et al., 2007). In addition, diet rich in betaine is associated with higher vaginal *Lactobacillus* spp. abundance (Tuddenham et al., 2019). Interestingly, a recent study by Song et al. (2020) reported that overall vaginal microbial diversity was higher among vegetarian women than non-vegetarians, although the sample size was small. Brookheart et al. (2019) reported that independent of ethnicity, overweight and obese women suffer more frequently from BV than lean women. Potential underlying reasons include obesity-associated dysfunction/disturbances in the host metabolism, hormonal, and immune system regulation which may affect vaginal environment. Furthermore, obesity is associated with dysbiotic gut microbiota composition which can further affect VMB composition by serving as an “extravaginal source” for vaginal bacteria (Marrazzo et al., 2012). However, larger studies exploring the role of various diets and/or body mass index (BMI) on specific CSTs are lacking.

Urbanization may also play a role in affecting the VMB composition by increasing diversity, but the data is yet limited to a few ethnic groups (Vargas-Robles et al., 2020). Among the socioeconomic factors, there is an indication that the education level seems to be associated with VMB composition. A study by Virtanen et al. (2019) showed that Finnish women aged 25–45 with higher education level had more often *Lactobacillus*-dominated VMB community (especially *L. crispatus*).

#### Immune System

Vaginal mucosal immune system both interacts with and regulates VMB composition [e.g., reviewed by Smith and Ravel (2017); Villa et al. (2020)]. The interplay between the immune system and the VMB is complex and involves variable factors such as epithelial and immune cells, antimicrobial peptides, pro/anti-inflammatory cytokines/chemokines, as well as secretory antibodies (Wira et al., 2010; Yarbrough et al., 2015). Epithelial and immune cells (dendritic cells) in the cervicovaginal mucosa maintain homeostasis with the VMB and simultaneously survey for pathogens. These cells detect microbial structures (antigens) *via* pattern recognition receptors such as Toll-like receptors (TLRs), which induces production of antimicrobial peptides as well immunomodulatory cytokines/chemokines. Dendritic cells further function as an integral link between innate and adaptive immune system by presenting antigens to other immune cells such as macrophages, NK-cells, neutrophils, and T- and B-cells (White et al., 1997; Duluc et al., 2013). In general, when epithelial cells encounter endogenous vaginal lactobacilli, epithelial cells produce low levels of antimicrobial peptides/cytokines resulting in mucosal homeostasis. Depletion of the endogenous lactobacilli and the dominance of BV-associated community results in higher levels and a different secretion profile of antimicrobial peptides as well as inflammatory cytokines/chemokines [reviewed by Smith and Ravel (2017), Yarbrough et al. (2015)]. Specifically, there are indications that vaginal lactobacilli assigned to CSTs affect differently the type and magnitude of innate immune response. For instance, there are reports showing that CST-IV induces higher pro-inflammatory response (IL-1α, IL-1β, TNF- α, IFN-γ, IL-4, IL-8, IL-12p70) than CST-I or CST-II, whereas CST-III induces an intermediate response (IL-8) (Rose et al., 2012; Anahtar et al., 2015). However, more studies are needed to understand in-depth how different CSTs regulate the immune system in a healthy state and transition into dysbiosis.

#### Vaginal Infections and Disorders/Dysbiosis and Antimicrobial Therapy

Bacterial vaginosis is the most common vaginal disorder/dysbiosis of women affecting at least 30% of women in reproductive age annually (Koumans et al., 2007; Bradshaw et al., 2013). BV is characterized by a decrease in lactobacilli and increase in atypical anaerobic bacteria (van de Wijgert and Jespers, 2017). BV increases the risk of the acquisition of sexually transmitted diseases, such as *Chlamydia trachomatis*, *Neisseria gonorrhoeae*, *Trichomonas vaginalis*, herpes simplex virus type 2 (HSV-2), HPV, and HIV (Cherpes et al., 2003; Wiesenfeld et al., 2003; Myer et al., 2005; Allsworth et al., 2008; Brotman et al., 2010; Liang et al., 2019). BV is also a risk factor for other infections such as pelvic inflammatory disease, endometritis, chorioamnionitis, and amniotic fluid infection (Hay et al., 1994; Hillier et al., 1995; Koumans et al., 1999). *C. albicans*-associated VVC is one of the most common mucosal infections in the female genital tract (De Bernardis et al., 2018). Estimations show that majority of women (>75%) suffer from *Candida* infections at least once in their lifetime (Bongomin et al., 2017). Some women are more susceptible to VVC and recurrence of VVC is common (Blostein et al., 2017). *C. glabrata* is the second most common type causing 15% of the infections (Shaaban et al., 2013).

The major modulators of VMB composition are antibiotics and antifungal medicines targeted against vaginal infections. Metronidazole and clindamycin are the first-line antibiotic regimens for BV. The short-term cure rates are approximately 80%, with 50% recurrence rate within 6–12 months (Bradshaw and Sobel, 2016). Biofilm formation as well as antibiotic resistance of BV-associated bacteria such as *G. vaginalis* may be determinant factors for persistence and recurrence (Bradshaw and Sobel, 2016; Verwijs et al., 2020). The same applies for *C. albicans* which is effective in biofilm formation (Chandra et al., 2001). The hyphal (mycelial) form contributes to adherence and mucosal invasion is characteristic of symptomatic disease (Pappas et al., 2018). Biofilm provides increased virulence as well as resistance to host immune responses’ antimicrobial agents and may lead to recurrent candidiasis and reduced impact of antifungal treatments (Bradford and Ravel, 2017; Kalia et al., 2020). With regard to endogenous lactobacilli, some trials show that use of metronizadole, doxycycline, azithromycin, clotrimazole, and fluconazole has no substantial impact on overall vaginal *Lactobacillus* spp. colonization (Agnew and Hillier, 1995; Nyirjesy et al., 2006). Specific vaginal lactobacilli tolerate metronidazole up to 1000 μg/ml *in vitro* (Simoes et al., 2001). Furthermore, in women with diagnosed BV, few studies show that VMB seems to recover from metronidazole treatment (metronidazole) within a few days (Mayer et al., 2015; Lehtoranta et al., 2020), and several studies show that *L. iners* dominates the community in transition to the recovery (Macklaim et al., 2015; Mayer et al., 2015; Deng et al., 2018; Lehtoranta et al., 2020).

#### Probiotics

Probiotics are defined as “live microorganisms that, when administered in adequate amounts, confer a health benefit on the host” (Hill et al., 2014). As probiotics are commonly lactobacilli, their role in vaginal health has been extensively investigated especially in the context of vaginal infections in premenopausal women (Borges et al., 2014; Petrova et al., 2015). Increasing evidence show that specific probiotic strains or their combinations elevate vaginal lactobacilli counts in healthy women or women with BV and/or VVC and support natural VMB during/after recovery from antibiotics/antifungal treatment (Xie et al., 2017; Li et al., 2019). However, there is high heterogeneity among the studies due to variation in study designs and probiotic strains used. Probiotics may elicit beneficial effects in the vaginal tract in several ways, i.e., by producing lactic acid and hydrogen peroxide to lower the vaginal pH. Furthermore, probiotics may produce antimicrobial compounds and stimulate the immune system to help to maintain bacterial balance in the vaginal tract. By adhering to vaginal epithelia, probiotics may inhibit attachment of pathogenic bacteria and utilize the same nutrients as pathogens and thereby restrict their growth (Petrova et al., 2015; Chee et al., 2020).

Probiotics targeted for vaginal health are widely available as dietary supplements or vaginal capsules/suppositories. In vaginal applications, probiotics are applied directly at the site of action, whereas orally supplemented probiotics need to first passage through the gastrointestinal tract before migrating to the vaginal tract. Interestingly, research shows that both application routes are efficacious (Li et al., 2019; Wang et al., 2019). Orally administered probiotics, however, may provide additional beneficial effects to vaginal health *via* so called “gut-vagina axis” by balancing gut microbiota and inhibiting/preventing ascension of urogenital pathogens from the rectum to vaginal tract as well as stimulating the gut and systemic immune system.

## Vaginal Microbiota Changes Across the Life Span and Impact in Health

**FIGURE 2:**
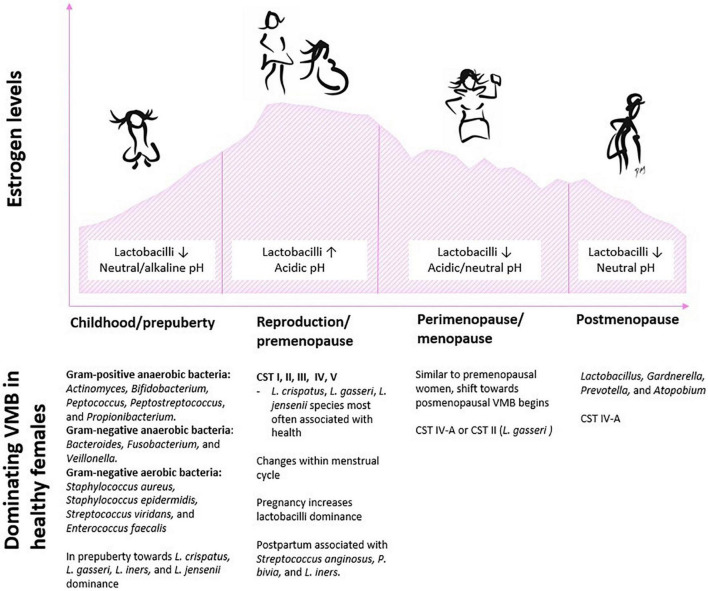
Schematic illustration of typical characteristics and an overview of dominating bacteria in the vaginal environment in context with fluctuating estrogen levels across the female life span. Abbreviations: CST, community state type; VMB, vaginal microbiota.

The VMB composition changes considerably across the female life span from childhood to the onset of puberty, during reproduction and pregnancy, as well over the transition period to menopause and after menopause (Figure 2). Below we take a closer look at the VMB composition at each lifestage and review available probiotic intervention studies conducted at each stage and summarize the key findings in Table 1. The primary focus here is on bacteria, since very little is known about the development of healthy vaginal mycobiota throughout life.

**TABLE 1:** Summary of potential benefits of probiotic intervention studies on vaginal microbiota (VMB) throughout female life span.

Childhood/puberty/adolescence	Fertility	Pregnancy	Menopause/postmenopause
- Lack of probiotic studies to conclude potential benefits in these age groups	- Potential to balance VMB composition, but findings yet inconclusive	- No significant changes in overall VMB composition reported - Some benefits in Group B *Streptococcus* colonization - No safety concerns - Additional potential benefits include - Balancing gut microbiota - Supporting metabolic health - Postpartum well-being	- Potential to balance and maintain healthy VMB composition as evidenced by - Increase in vaginal lactobacilli levels after consumption/application - Nugent score between 0 and 3 - Reduction of the risk of vaginal infections

### Childhood and Puberty/Adolescence

During perinatal development, residual maternal estrogen induces thickening of the vaginal epithelium and glycogen deposit in the epithelial cells. When epithelial cells exfoliate, glycogen is released, which favors glucose-fermenting micro-organisms. After birth, the maternal estrogen is metabolized, resulting in a thinning of the mucosa and reduction in glycogen- and glucose-fermenting microorganisms, and leads to dominance of wide range of aerobes and facultative anaerobes [reviewed by Younes et al. (2018)]. In childhood, VMB is dominated by Gram-positive anaerobic bacteria (including species from genera *Actinomyces, Bifidobacterium, Peptococcus, Peptostreptococcus*, and *Propionibacterium*), Gram-negative anaerobic bacteria (such as species from genera *Bacteroides, Fusobacterium*, and *Veillonella*), as well as aerobic bacteria (such as *Staphylococcus aureus, Staphylococcus epidermidis, Streptococcus viridans*, and *Enterococcus faecalis*) (Hammerschlag et al., 1978a,b; Myhre et al., 2002; Ranđeloviė et al., 2012). The vaginal pH of a young girl changes from birth until pre-puberty to become neutral or slightly alkaline [reviewed by Farage and Maibach (2006)].

Prepubertal girls have low abundance of lactobacilli, *G. vaginalis*, and *Prevotella bivia* in the vaginal tract (Myhre et al., 2002; Ranđeloviė et al., 2012). In the onset of puberty, when estrogen levels increase, the vaginal epithelium thickens, which selectively favors glucose-fermenting microorganisms. The microbiome of adolescent girls resembles VMB of adult women and is dominated by *L. crispatus, L. gasseri, L. iners*, and *L. jensenii* (Yamamoto et al., 2009; Thoma et al., 2011a; Hickey et al., 2015). Furthermore, the role of genetic heritage on VMB composition has been addressed by collecting vaginal samples from the mothers (Hickey et al., 2015). However, no consistent correlation exists in the VMB composition similarity between mothers and their daughters. Therefore, the role of genetic component in VMB composition remains unclear.

The most common cause of gynecological complaints in children and young girls is vulvovaginitis [reviewed by Beyitler and Kavukcu (2017)]. Risk factors include hypoestrogenism, anatomical proximity of rectum, and delicate vulvar skin and vaginal mucosa. Research suggests that the main causative agents for prepubertal vulvovaginitis are of fecal origin, such as *Escherichia coli* and *E. faecalis* (Bumbulienė et al., 2014) or Group A beta-hemolytic streptococci (Gerstner et al., 1982; Ranđeloviė et al., 2012; Bumbulienė et al., 2014). The clinical features, however, drive the interpretation whether an isolated microorganism is part of the normal VMB or is the cause of symptomatic vulvovaginitis. In contrast in puberty, *C. albicans* seems to be the most prevalent micro-organism isolated in vulvovaginitis infections (Yilmaz et al., 2012). As elevated levels of estrogen concentrations and subsequent glucose in the vaginal epithelium during the pubertal period increase susceptibility to *C. albicans*, it is logical that *C. albicans* vaginal infections are less prevalent in pre-puberty.

Investigation of VMB in adolescents may provide insight into the complexity and variability of VMB in adults and may help to understand whether the VMB development during puberty is associated with vaginal health in adulthood. Most likely, physiologic changes occurring in puberty and initiation of cyclic menstruation have profound effects on VMB, but research during these stages is very limited.

#### Probiotics

Thus far, no reports exist on probiotics and their impact on VMB in pre-puberty or puberty. However, it is important to acknowledge the role of estrogen in driving the VMB composition in pre-puberty and puberty, and this should be taken into consideration when designing probiotic studies in this age group for supporting vaginal health.

### Reproduction and Fertility

#### Fertility

Infertility is a global concern affecting millions of people of reproductive age (Mascarenhas et al., 2012). In women, multiple factors can cause infertility, such as disorders concerning fallopian tubes, uterus, ovaries, as well as the endocrine system (Walker and Tobler, 2021). Knowledge on the role of VMB in fertility is increasing. BV affects, on average 30% of women in reproductive age and is a known risk factor for infertility [Koumans et al., 2007; reviewed by van Oostrum et al. (2013)]. Research shows that, particularly, the depletion of vaginal lactobacilli coupled with increased presence of *G. vaginalis*, *A. vaginae*, *Ureaplasma parvum*, *Ureaplasma urealyticum*, as well as *Candida* spp. are associated with issues in fertility [reviewed by Koedooder et al. (2019a)]. Vaginal dysbiosis is also linked with increased risk of sexually transmitted diseases (STDs) and may promote ascension of bacterial pathogens to fallopian tubes affecting reproduction (García-Velasco et al., 2017, 2020). Low abundance of vaginal lactobacilli is associated with infertility and vice versa (García-Velasco et al., 2020; Hong et al., 2020). Furthermore, composition of VMB can impact the success of fertility treatment/artificial reproductive technologies. For instance, high dominance of vaginal lactobacilli and in particular *L. crispatus* seems to affect positively on a successful outcome of the *in vitro* fertilization/embryo transfer (Hyman et al., 2012; Koedooder et al., 2019b).

##### Probiotics

Probiotics represent an attractive means to balance microbiota and counteract dysbiosis of the reproductive tract thus positively influencing fertility. Probiotic lactobacilli could be used to “prime” the VMB in the pre- and peri-conceptual periods and to prevent pathogenic infections associated with fertility and PTB. However, current research on the role of probiotics in fertility and/or infertility is yet scarce and inconclusive (Corbett et al., 2021). In an open label clinical trial with 117 women, a single dose of a vaginally administered probiotic containing *Lactobacillus acidophilus, Bifidobacterium bifidum*, and *Bifidobacterium longum* at the time of egg retrieval for *in vitro* fertilization (IVF) was not associated with an increase in vaginal *Lactobacillus* colonization or with improved pregnancy outcomes (Gilboa et al., 2005). In another study, *Ligilactobacillus salivarius* CECT5713 (formerly *Lactobacillus salivarius*) was administered daily for 6 months to 44 pregnant women either with a history of recurrent miscarriages or infertility with abnormal VMB and vaginal pH at baseline. The intervention resulted in 56% of successful pregnancy rate in these women and those with full-term pregnancy had significant increase in vaginal lactobacilli, decrease in vaginal pH, and Nugent score after intervention, suggesting probiotic’s ability to balance VMB and support healthy pregnancy in women with fertility issues (Fernández et al., 2021). Women with unexplained infertility diagnosis and receiving a 4-week probiotic supplementation of *L. crispatus* LbV 88, *L. rhamnosus* LbV 96, *L. jensenii* LbV 116, and *L. gasseri* LbV 150N had no change in overall VMB diversity or composition when compared with controls (Schenk et al., 2021). Interestingly, after the intervention, the relative abundance of *Ureaplasma parvum*, a pathogen associated with infertility and reproduction issues, was significantly lower in the probiotic group (0.77% vs. 3.52%, probiotic vs. control).

In the future, carefully designed and adequately powered clinical trials with well-characterized probiotic strains and treatment regimens are necessary to evaluate the effect of probiotics on fertility outcomes (Mohapatra et al., 2019; Balaghi et al., 2020; Cozzolino et al., 2020).

#### Pregnancy

In normal healthy pregnancy, studies comparing VMB composition between pregnant and non-pregnant women show that lactobacilli dominance increases throughout pregnancy along with increased stability and decreased dominance of bacteria associated with BV (e.g., Romero et al., 2014; Serrano et al., 2019). Notably, VMB change and increase in lactobacilli, especially *L. iners*, throughout pregnancy is most pronounced in women of African ancestry (Serrano et al., 2019). Among dominating lactobacilli, *L. crispatus*, seems to be the most stable species across pregnancy (Serrano et al., 2019). These findings are supported by several studies [reviewed by Anahtar et al. (2018)]. After delivery in the postpartum period, the composition of VMB shifts to less diverse and is characterized by lower abundance of *Lactobacillus* spp. (CST I, II, CST V) and significantly higher proportions of *Streptococcus anginosus*, *P. bivia*, and *L. iners* (Nunn et al., 2021).

It is likely that both estrogen and progesterone contribute to the increased dominance of lactobacilli during pregnancy, which stimulate glycogen accumulation in the vaginal epithelial cells favoring *Lactobacillus* spp. colonization (Anahtar et al., 2018). During pregnancy, the function of the mucosal barrier decreases due to congestion and edema of vaginal mucosa. Furthermore, pregnancy increases immune system modulation, which may lead to differential responses against pathogens (Mor and Cardenas, 2010). Microbial dysbiosis is linked with negative reproductive outcomes, such as prelabor rupture of membranes and PTB (Elovitz et al., 2019). Fettweis and colleagues assessed how VMB changes across pregnancies correlate with the risk of PTB by comparing data from 45 preterm and 90 term birth controls in a cohort of women of predominantly African ancestry (Fettweis et al., 2019). Those women who delivered preterm significantly exhibited lower vaginal levels of *L. crispatus* and, e.g., higher levels of BVAB1, *Sneathia amnii*, and *Prevotella* spp. Early pregnancy, such as the first 8 weeks of gestation, is a critical time for successful pregnancy as most miscarriages occur early in the first trimester (Preisler et al., 2015). A study by Al-Memar et al. (2020) investigating 161 pregnancies reported that in miscarriages occurring during the first trimester, the vaginal bacterial composition was less abundant with *Lactobacillus* spp., and a significantly higher proportion of these cases were dominated by CST IV.

##### Probiotics

Probiotics could provide natural means to support healthy and balanced VMB composition throughout pregnancy and reduce the risk of microbial dysbiosis. Thus far, clinical studies in pregnant women targeting VMB have examined either probiotic effects on overall VMB composition or specifically on Group B *Streptococcus* (GBS) colonization.

The studies assessing probiotic effects on VMB composition or changes throughout pregnancy have used either oral or vaginal probiotics. Oral administration of *Lacticaseibacillus rhamnosus* GR-1 and *Limosilactobacillus reuteri* RC-14 either from 9 to 14 weeks of gestation to delivery (Husain et al., 2020) or for 8 weeks in mid-pregnancy (Gille et al., 2016) did not have a significant impact on VMB as assessed by Nugent score when compared with placebo (Gille et al., 2016; Husain et al., 2020). Similarly, supplementation with the combination of species of *Bifidobacterium longum*, *Lactobacillus delbrueckii* subsp. *bulgaricus*, and *Streptococcus thermophilus* from 32 weeks of gestation until delivery had no significant changes in VMB composition when analyzed by 16S and compared with controls (Liang et al., 2021). In a study by Stojanović et al. (2012), the effect of vaginal application containing *L. rhamnosus* BMX 54 once a week for 12 weeks was assessed in pregnant women enrolled at second trimester of their pregnancy. The women receiving vaginal probiotic had more stable VMB and did not experience significant changes over the course of the study, whereas women without probiotics had a significant trend toward increase in the presence of vaginal pathogenic microorganisms, especially *C. albicans* over the course of the study.

Maternal GBS colonization is a predominant risk factor for both early- and late-onset neonatal sepsis. Regardless of intrapartum antibiotic prophylaxis (IAP), infants of GBS-colonized and antibiotic-treated mothers are at some risk of presenting with sepsis [reviewed by Patras and Nizet (2018)]. Probiotics with their antimicrobial potential could be used to support healthy gut and VMB and thus reduce GBS colonization. No impact on GBS colonization was found with consumption of four-strain probiotic blend *L. jensenii* Lbv116, *L. crispatus* Lbv88, *L. rhamnosus* Lbv96, and *L. gasseri* Lbv150 when consumed for 2 weeks in GBS-positive women with 33–37 weeks of gestation (Farr et al., 2020). Furthermore, no significant impact on GBS colonization was observed in an open-label trial of 10 women with a probiotic combination containing *L. acidophilus* strains La-14 and NCFM, *Bifidobacterium animalis* subsp. *lactis* strains HN019 and Bi-07, and *B. longum* subsp. *longum* Bl-05 either, when taken from 28 weeks until 36 weeks of pregnancy (Hanson et al., 2014). The efficacy of vaginal isolate *L. salivarius* CECT 9145 against intestinal and vaginal GBS eradication was tested in a pilot trial involving 57 healthy GBS-positive pregnant women from week 26 to week 38 (Martín et al., 2019). At the end of the intervention, there was reduction in the rate of GBS-colonized women receiving *L. salivarius* which further decreased the number of women receiving IAP during delivery. Olsen et al. showed that oral probiotics *L. rhamnosus* GR-1 and *L. reuteri* RC-1 when taken either at mid-pregnancy or 3 weeks prior to delivery had no significant impact on GBS rates when compared with controls (Olsen et al., 2018; Sharpe et al., 2019). However, in another study, the same combination was taken at 35–37 weeks of pregnancy until delivery showed that 42.9% of the GBS-positive women in the probiotic group compared with 18.0% in the placebo group achieved a negative GBS culture at the time of delivery (Ho et al., 2016).

Taken together, the above studies indicate that the VMB of women in healthy pregnancy is relatively stable and probiotics as such may not induce significant changes on the VMB composition. Larger and well-controlled trials are needed to make further conclusions on their efficacy. Nevertheless, probiotics do not negatively impact VMB, and no safety concerns have been raised with their use in pregnancy (Sheyholislami and Connor, 2021). Furthermore, probiotics may have other beneficial effects in pregnant women, such as balancing the gut microbiota and having a beneficial effect on metabolic outcomes, such as gestational diabetes mellitus (Lv et al., 2018) and postpartum depression/anxiety (Slykerman et al., 2017). It should be noted that probiotic effects are strain-specific and the dose, stability, length of intervention, delivery vehicle/matrix, and survival in the gut and vaginal tract are essential for their ability to exert positive health effects.

### Menopause and Postmenopause

As the hormonal balance shifts significantly during menopausal transition, changes are also evident in the VMB of postmenopausal women. The most dominant vaginal bacterial species in healthy postmenopausal women are those from genera *Lactobacillus*, *Gardnerella, Prevotella*, and *Atopobium* (Shen et al., 2016). The percentage of *Lactobacillus* spp. has shown to be lower after menopause compared to pre- and perimenopausal period (Hillier and Lau, 1997; Brotman et al., 2014b; Kim et al., 2021). A cross-sectional study by Brotman et al. (2014b) demonstrated that the abundance of *Lactobacillus* spp. in the vagina of pre- and perimenopausal women was 83%, whereas the abundance of those in postmenopausal women was 54%. In the same study, it was shown that menopausal status and CST strictly correlate with each other: perimenopausal women were most often classified to have type CST IV-A or the *L. gasseri* CST, whereas postmenopausal women were typically CST IV-A, a type with large abundance of anaerobic bacteria from the genera *Anaerococcus*, *Peptoniphilus*, *Prevotella*, and *Streptococcus* (as compared to premenopausal women with CSTs dominated by *L. crispatus* and *L. iners*).

The use of hormone replacement therapy (HT) has been shown to affect VMB in postmenopausal women (Pabich et al., 2003; Devillard et al., 2004; Gliniewicz et al., 2019). A study by Gliniewicz et al. (2019) including both pre- and postmenopausal women showed that the vaginal CST of postmenopausal women under HT was more likely to resemble that of premenopausal women than that of postmenopausal women without HT. These findings emphasize the role of estrogen in defining the VMB composition. However, the association between clinical vulvovaginal symptoms and *Lactobacillus* levels is not so clear. According to Mitchell et al. (2017), despite the *Lactobacillus* spp. dominance, the number of vaginal symptoms was not any less, whereas other studies have reported that vaginal lactobacilli abundance and the symptoms are associated inversely in postmenopausal women (Shen et al., 2016). Furthermore, another study by Mitchell et al. (2018) showed some indications (not reaching the level of significance) that the potential to improve genitourinary symptoms is more pronounced with higher lactobacilli abundance.

The decrease in the vaginal *Lactobacillus* abundance in postmenopausal women can lead to higher microbial diversity and higher vaginal pH increasing the risks for infections (Ginkel et al., 1993; Gliniewicz et al., 2019).

Menopause-related decline in estrogen (17β-estradiol) levels is associated with changes in VMB and subsequently in the (vulvo)vaginal lining. Vulvovaginal atrophy (VVA) is a common condition, affecting 25–50% of postmenopausal women, causing various symptoms such as itching, discharge, bleeding, burning sensations, and difficulties in intercourse (Bachmann and Nevadunsky, 2000). Brotman et al. (2014b) demonstrated that VMB is associated with the signs of VVA: postmenopausal women with type CST IV-A VMB were most prone to VVA compared to pre- and perimenopausal women. Shen et al. (2016) demonstrated similarly that postmenopausal women with atrophic vaginitis (AV) have lower abundance in lactobacilli than healthy postmenopausal women. According to their results, genera *Gardnerella* and *Atopobium* were related to AV after menopause.

The increased vaginal pH along with declining ovarian estrogen during menopause allows the growth of harmful microbes such as *Escherichia coli*, *Candida* spp., and *Gardnerella* spp. leading to increased risk of BV and VVC [reviewed by Kim and Park (2017)]. The diagnosis of BV and thus defining VMB composition among postmenopausal women has been considered challenging, since many of the presumptions related to the testing procedures are only valid for premenopausal/fertile women (Cauci et al., 2002). Firstly, vaginal pH is commonly increased at menopause, whereas during fertile years, it is an indication of BV (according to Amsel criteria). Secondly, what comes to Nugent scoring method, it assumes that normal VMB is highly abundant with lactobacilli, which is not the case among postmenopausal women. In a study including pre-, peri-, and postmenopausal women, Cauci et al. (2002) showed, that in postmenopausal women with low lactobacilli abundance, BV prevalence was lower compared to fertile or perimenopausal women. It has been suggested that instead being a sign of BV, higher vaginal pH, in the absence of pathogenic bacteria, is an indication of menopause. To support this theory, it has been shown that serum estradiol levels are negatively associated with the vaginal pH (Caillouette et al., 1997).

Vulvovaginal candidiasis is considered to be strongly associated with estrogens; therefore, the prevalence of *Candida* spp. seems to decrease with aging (Hoffmann et al., 2014). However, VVC as a possible adverse effect of estrogen-based HT is a common finding (Nwokolo and Boag, 2000; Fischer and Bradford, 2011). Furthermore, postmenopausal women with uncontrolled chronic conditions such as diabetes or immunosuppressive disorders, are specifically prone for recurrent complicated VVCs (Nwokolo and Boag, 2000). This susceptibility is mainly due to already compromised immune status and possible excessive use of antibiotics creating opportunities for *Candida* spp. invasion (Low and Rotstein, 2011).

#### Probiotics

As mentioned above, Nugent score is one of the key diagnostic tools for BV. Intermediate Nugent score (4–6) is of particular interest as it is seen as a risk for developing BV (Guedou et al., 2012; Amegashie et al., 2017). A study by Petricevic et al. (2008) showed that VMB can be balanced in postmenopausal women (*n* = 72) with intermediate Nugent score by 14-day supplementation of probiotics *L. rhamnosus* GR-1 and *L. reuteri* RC-14. In this clinical trial, Nugent score improved in 60% of the women in the probiotic group, whereas the improvement was only 16% in the placebo group. Another study with the same strains demonstrated that 3-day vaginal administration of the probiotics did not change the Nugent score in postmenopausal women (*n* = 14) with intermediate Nugent scores (Bisanz et al., 2014). However, the 16S sequencing analyses showed that there was a significant increase in the vaginal abundance of *Lactobacillus* spp. in the probiotic group, indicating a potential health benefit from the microbiota perspective.

Promising results have been received from a clinical trial performed in 22 menopausal breast cancer patients under chemotherapy (Marschalek et al., 2017). The study participants had vaginal atrophy and intermediate Nugent Score at the baseline and received either combination of four probiotics (*L. crispatus* LbV 88, *L. rhamnosus* LbV 96, *L. jensenii* LbV 116, *L. gasseri* LbV 150N) or placebo twice a day for 2 weeks. Vaginal swabs were collected at baseline, 1 day after the treatment, and 1 week after the treatment. Improvement toward healthy Nugent Score (0–3) was detected in 63% women in the probiotic group and in 36% in the placebo group. Nugent Score was reduced at Day 1 and after 1 week in the probiotic group. In addition, a pilot placebo-controlled clinical trial by Kwak et al. (2017) investigated the vaginal colonization of topically applied *L. gasseri* LN40, *Limosilactobacillus fermentum* LN99, and *L. rhamnosus* LN113 product for 10 days in postmenopausal women (*n* = 18). Potential vaginal persistence was shown for LN99 and LN113 either at the end or after the intervention, but not for LN40. Another study in postmenopausal women with symptoms of vaginal atrophy and taking vaginal tablets containing low dose of estriol (0.03 mg) and *L. acidophilus* KS400 showed benefits in improving vaginal maturation index and related clinical symptoms (Jaisamrarn et al., 2013).

Furthermore, there are multiple studies on BV and probiotic benefits on VMB, including women with the age range varying from 18 to 70 years [reviewed by Hanson et al. (2016); Li et al. (2019), Wang et al. (2019); van de Wijgert and Verwijs (2020)]. In most of the studies there is no clear differentiation between pre- and postmenopausal women. An observational pilot study in women with history of recurrent BV and undergoing surgical menopause showed that when *L. rhamnosus* BMX 54 was administered as vaginal tablets altogether for 6 months, women experienced less BV recurrences (Parma et al., 2013). However, due to relatively low number of studies focusing on menopausal/postmenopausal population only, more studies on probiotics’ effects on VMB focusing on this population are warranted.

## Conclusion and Future Perspectives

As research evidence accumulates, it is apparent that VMB and especially vaginal lactobacilli play a crucial role in women’s health throughout life. In healthy women, estrogen levels are one of the major factors shaping the VMB from the onset of puberty to menopause and postmenopause (Figure 2) as well as across menstrual cycle and pregnancy. Furthermore, ethnicity and variable external factors such as lifestyle and antimicrobial medication are well-known to determine and influence the composition and dysbiosis/homeostasis state. Through rapid technology development, research is starting to unravel the interactions between the VMB and the human host and how the microbial balance shifts from homeostasis to dysbiosis and progresses to disease. Nevertheless, the impact of balanced vaginal bacterial as well fungal community on health is not fully characterized, and research has mainly focused on these organisms’ associations with disease. Especially, the role of vaginal fungi has been neglected although evidence indicates that mycobiota is important and, for example, gut-associated fungi can modulate host immune responses (Underhill and Iliev, 2014; Wheeler et al., 2016; Raimondi et al., 2019). Symbiosis exists between yeast and bacteria in different environmental niches, which applies also in the vaginal tract. Polymicrobial interactions (Krüger et al., 2019) and factors turning a normal fungal inhabitant as a pathogenic organism (Sam et al., 2017) have received attention in the past years. However, more research is necessary to elucidate bacteria–fungi and fungi–fungi relationships and interactions. Understanding of these interactions as well as signaling and immunological responses together with metabolites produced by bacteria and fungi in vaginal health and disease will open new means for microbiota management. For example, for BV, alternative options for its management are much needed, as the pathology is still not well-understood, and the current treatment options are inadequate in efficacy and the recurrence rate is high.

Probiotics are considered as natural and safe means to balance the VMB composition and support during antimicrobial treatment or help in recovery from dysbiosis. However, although safety of probiotics has been well-established, not all clinical studies have been successful in terms of efficacy, and large variation exists between the studies in terms of probiotic strains/combinations used, the target population by ethnicity and age/life stage, and study designs. Furthermore, there is an increasing interest to understand how probiotics support women’s health throughout life and how to take these specific conditions into account (and especially the hormonal fluctuations), when designing probiotic products and studies for women. Research should also focus on identifying the most optimal probiotic species/strains for vaginal health and VMB composition and their mechanisms of actions on VMB and the host. In the future, indigenous vaginal lactobacilli consortia and/or transplantations of healthy women may be more effective solutions for women suffering from vaginal dysbiosis and associated infections/adversities. For example, fecal microbiota transplantation has been successfully applied to treat *Clostridioides difficile* infections. Indeed, a similar approach has been applied to treat recurrent intractable BV (Lev-Sagie et al., 2019), where promising results were reported in terms of achieving long-term remission of BV after 5–12 months of VMB transplantation from healthy donors.

## Author Contributions

All authors wrote the original draft of the manuscript, contributed to the article, and approved the submitted version.

## Conflict of Interest

LL, RA-J, AL, and JM are current or previous workers of International Flavors and Fragrances, a company that manufactures probiotics.

## Publisher’s Note

All claims expressed in this article are solely those of the authors and do not necessarily represent those of their affiliated organizations, or those of the publisher, the editors and the reviewers. Any product that may be evaluated in this article, or claim that may be made by its manufacturer, is not guaranteed or endorsed by the publisher.
